# Supplementary material for: Alterations of the Composition and Neurometabolic Profile of Human Gut Microbiota in Major Depressive Disorder
Source: Biomedicines. 2022 Sep 2;10(9):2162. doi: 10.3390/biomedicines10092162 (PMC9496097; doi:10.3390/biomedicines10092162)
Supplement: Supplementary file 1 [file biomedicines-10-02162-s001.zip › Supplementary Table S3.pdf]

Supplementary Table S3. The structure of the catalog of orthologs.

Enzyme name	Number of orthologs	Bacterial origin (genus)	Function
2-oxoisovalerate dehydrogenase alpha	5	*Enterococcus, Lactobacillus, Listeria, Pseudomonas, Streptococcus*	Isovaleric acid synthesis (KADH pathway)
2-oxoisovalerate dehydrogenase beta	5	*Enterococcus, Lactobacillus, Listeria, Pseudomonas, Streptococcus*	Isovaleric acid synthesis (KADH pathway)
Aldehyde dehydrogenase	10	*Bacteroides, Clostridium, Coprococcus, Corynebacterium, Eggerthella, Escherichia, Eubacterium, Listeria, Pseudomonas, Streptococcus*	Isovaleric acid synthesis (KADH pathway)
Pyruvate decarboxylase	1	*Escherichia*	Isovaleric acid synthesis (KADC pathway)
4-aminobutyrate aminotransferase gabT	12	*Anaerostipes, Bifidobacterium, Blautia, Citrobacter, Clostridium, Coprococcus, Enterobacter, Escherichia, Eubacterium, Klebsiella, Roseburia, Streptococcus*	GABA degradation
4-aminobutyrate aminotransferase PuuE	4	*Citrobacter, Enterobacter, Escherichia, Klebsiella*	GABA degradation
4-cresol dehydrogenase	1	*Pseudomonas*	p-Cresol degradation
Protocatechuate 3,4-dioxygenase pcaG	7	*Citrobacter, Enterobacter, Escherichia, Klebsiella, Proteus, Pseudomonas, Streptococcus*	p-Cresol degradation
Protocatechuate 3,4-dioxygenase pcaH	7	*Citrobacter, Enterobacter, Escherichia, Klebsiella, Proteus, Pseudomonas, Streptococcus*	p-Cresol degradation
4-hydroxyphenylacetate decarboxylase hpdB	8	*Alistipes, Bacteroides, Bacteroides, Clostridioides, Escherichia, Lactobacillus, Roseburia, Ruminococcus*	p-Cresol synthesis
4-hydroxybutyrate dehydrogenase	13	*Alistipes, Anaerostipes, Bifidobacterium, Blautia, Citrobacter, Clostridium, Dorea, Enterobacter, Escherichia, Eubacterium, Megasphaera, Roseburia, Ruminococcus*	γ -hydroxybutyric acid degradation
4-hydroxyphenylacetate 3-monooxygenase	7	*Citrobacter, Enterobacter, Escherichia, Klebsiella, Proteus, Providencia, Streptococcus*	Degradation of aromatic compounds (4-hydroxyphenylacetate)
Acetylserotonin O-methyltransferase	8	*Bacillus, Bacteroides, Chromobacterium, Clostridium, Desulfovibrio, Enterobacter, Pseudomonas*	Melatonin synthesis
Alanine racemase alr	6	*Citrobacter, Enterobacter, Escherichia, Klebsiella, Proteus, Providencia*	D-Alanine synthesis
Alanine racemase dadx	6	*Citrobacter, Enterobacter, Escherichia, Klebsiella, Proteus, Providencia*	D-Alanine synthesis
Argininosuccinate lyase	27	*Alistipes, Anaerostipes, Bacteroides, Blautia, Butyrivibrio,*	Arginine synthesis

		Citrobacter, Clostridium, Coprococcus, Desulfovibrio, Dialister, Dorea, Enterobacter, Enterococcus, Escherichia, Eubacterium, Faecalibacterium, Klebsiella, Lactobacillus, Odoribacter, Parabacteroides, Prevotella, Proteus, Providencia, Roseburia, Ruminococcus, Streptococcus	
Aromatic amino acid hydroxylases	7	*Chromobacterium, Enterobacter, Ferrimonas, Pseudomonas, Streptococcus, Vibrio, Vibrio*	Catecholamines' synthesis
Asparagine synthetase asnA	16	*Alistipes, Alistipes, Bacteroides, Bifidobacterium, Clostridium, Enterobacter, Enterococcus, Escherichia, Eubacterium, Faecalibacterium, Klebsiella, Lactobacillus, Prevotella, Proteus, Roseburia, Streptococcus*	Biosynthesis of asparagine
Asparagine synthetase asnB	7	*Alistipes, Bacteroides, Enterobacter, Enterococcus, Escherichia, Klebsiella, Prevotella*	Biosynthesis of asparagine
Aspartate aminotransferase	7	*Citrobacter, Enterobacter, Escherichia, Helicobacter, Klebsiella, Proteus, Providencia*	Kynurenine acid formation out of kynurenine
Butyrate kinase	28	*Acidaminococcus, Alistipes, Anaerotruncus, Bacteroides, Blautia, Butyrivibrio, Clostridium, Desulfovibrio, Enterococcus, Eubacterium, Faecalibacterium, Klebsiella, Lactobacillus, Listeria, Prevotella, Roseburia, Streptococcus*	Butyrate synthesis
Butyryl-CoA dehydrogenase	32	*Actinomyces, Alistipes, Anaerostipes, Blautia, Butyrivibrio, Citrobacter, Clostridium, Dialister, Dorea, Eggerthella, Enterobacter, Enterococcus, Escherichia, Eubacterium, Faecalibacterium, Fusobacterium, Gordonibacter, Helicobacter, Lactobacillus, Listeria, Megasphaera, Odoribacter, Peptoclostridium, Prevotella, Proteus, Providencia, Roseburia, Ruminococcus, Streptococcus*	Butyric acid synthesis
Carboxylesterase	6	*Citrobacter, Enterobacter, Escherichia, Klebsiella, Proteus, Streptococcus*	Benzoic acid synthesis
Carnitine dehydrogenase	2	*Enterobacter, Pseudomonas*	Utilization of L- and D-carnitine
Catalase	11	*Anaerococcus, Clostridium, Dorea, Enterococcus, Escherichia, Faecalibacterium,*	Antioxidant

		Helicobacter, Lactobacillus, Listeria, Proteus, Streptococcus	
Glutathione peroxidase	24	*Actinomyces, Alistipes, Bacteroides, Bifidobacterium, Butyrivibrio, Citrobacter, Clostridium, Dialister, Dorea, Enterobacter, Enterococcus, Escherichia, Eubacterium, Klebsiella, Lactobacillus, Lactococcus, Listeria, Megasphaera, Prevotella, Proteus, Providencia, Roseburia, Ruminococcus, Streptococcus*	Antioxidant
Superoxide dismutase SodA	15	*Alistipes, Bacteroides, Citrobacter, Clostridium, Desulfovibrio, Enterobacter, Enterococcus, Escherichia, Eubacterium, Klebsiella, Oxalobacter, Prevotella, Proteus, Providencia, Streptococcus*	Antioxidant
Superoxide dismutase SodB	12	*Alistipes, Bacteroides, Citrobacter, Enterobacter, Escherichia, Helicobacter, Klebsiella, Oxalobacter, Prevotella, Proteus, Providencia, Streptococcus*	Antioxidant
Superoxide dismutase SodC	11	*Citrobacter, Clostridium, Desulfovibrio, Enterobacter, Escherichia, Eubacterium, Helicobacter, Klebsiella, Proteus, Providencia, Streptococcus*	Antioxidant
Cell wall hydrolase P40	1	*Lacticaseibacillus*	Bacterial cell wall defense
Cell wall hydrolase P75	1	*Lactobacillaceae*	Bacterial cell wall defense
Chorismate mutase	8	*Actinomyces, Citrobacter, Clostridium, Enterobacter, Escherichia, Proteus, Providencia, Streptococcus*	Prephenate formation, part of phenylalanine and tyrosine synthesis pathways
Creatinine amidohydrolase	5	*Anaerostipes, Anaerotruncus, Clostridium, Clostridium, Pseudomonas*	Creatinine synthesis
Diacylglycerol kinase	8	*Citrobacter, Desulfovibrio, Enterobacter, Escherichia, Helicobacter, Proteus, Providencia, Streptococcus*	Phosphatidic acid and diacylglycerol synthesis
Dihydrolipoyl dehydrogenase	14	*Bacteroides, Bifidobacterium, Clostridium, Coprococcus, Dorea, Enterococcus, Escherichia, Eubacterium, Lactobacillus, Listeria, Pseudomonas, Ruminococcus, Streptococcus*	Regulatory protein (host-bacterium)
Dihydroxyacetone phosphatase	5	*Bacteroides, Bifidobacterium, Blautia, Clostridium, Corynebacterium*	Produce 1,3-dihydroxyacetone
D-lactate dehydrogenase	13	*Citrobacter, Clostridium, Desulfovibrio, Enterobacter, Enterococcus, Escherichia, Lactobacillus, Proteus,*	D-lactic acid formation

		Providencia, Streptococcus	
Dopa decarboxylase	10	*Bacillus, Bacteroides, Desulfovibrio, Desulfovibrio, Pseudomonas, Pseudonocardia, Rubrobacter, Streptococcus, Yersinia*	Serotonin, dopamine and norepinephrine synthesis
D-serine/D-alanine/glycine transporter	6	*Citrobacter, Enterobacter, Escherichia, Klebsiella, Proteus, Providencia*	Transportation of D-serine, D- alanine and glycine
Estradiol 17-beta-dehydrogenase	29	*Acidaminococcus, Actinomyces, Alistipes, Anaerofustis, Anaerostipes, Bacteroides, Blautia, Butyrivibrio, Citrobacter, Clostridium, Coprococcus, Dialister, Dorea, Enterobacter, Enterococcus, Escherichia, Eubacterium, Faecalibacterium, Klebsiella, Lactobacillus, Megamonas, Megasphaera, Oxalobacter, Prevotella, Proteus, Providencia, Roseburia, Ruminococcus, Streptococcus*	17-beta-Estradiol degradation
Ethanolamine ammonia-lyase eutB	10	*Citrobacter, Clostridium, Desulfovibrio, Enterobacter, Enterococcus, Escherichia, Klebsiella, Listeria, Proteus, Providencia*	Ethanolamine catabolism
Ethanolamine ammonia-lyase eutC	9	*Citrobacter, Clostridium, Enterobacter, Enterococcus, Escherichia, Klebsiella, Listeria, Proteus, Providencia*	Ethanolamine catabolism
Gamma-aminobutyrate antiporter	20	*Alistipes, Alistipes, Bacteroides, Bifidobacterium, Clostridium, Desulfovibrio, Enterobacter, Enterococcus, Escherichia, Eubacterium, Lactobacillus, Listeria, Megasphaera, Odoribacter, Parabacteroides, Prevotella*	GABA transportation
Gamma-glutamyltranspeptidase	12	*Acidaminococcus, Bacillus, Bacteroides, Citrobacter, Clostridium, Enterobacter, Escherichia, Helicobacter, Klebsiella, Proteus, Providencia, Streptococcus*	Glutathione degradation
Glutamate decarboxylase	28	*Alistipes, Bacteroides, Bifidobacterium, Bifidobacterium, Clostridium, Clostridium, Desulfovibrio, Eggerthella, Enterococcus, Escherichia, Eubacterium, Gordonibacter, Helicobacter, Lactobacillus, Lactococcus, Listeria, Odoribacter, Parabacteroides, Parvimonas, Prevotella, Proteus, Streptococcus*	GABA synthesis
Glutamate mutase glmE	8	*Bacteroides, Blautia, Citrobacter,*	Glutamate II degradation

		Clostridium, Enterobacter, Escherichia, Prevotella, Proteus	
Glutamate mutase glmS	8	*Bacteroides, Blautia, Citrobacter, Clostridium, Enterobacter, Escherichia, Prevotella, Proteus*	Glutamate II degradation
Methylaspartate ammonia-lyase	8	*Bacteroides, Blautia, Citrobacter, Clostridium, Enterobacter, Escherichia, Prevotella, Proteus*	Glutamate II degradation
Glutamate synthase gltB	11	*Anaerostipes, Bacillus, Bifidobacterium, Blautia, Clostridium, Escherichia, Lactobacillus, Listeria, Roseburia, Ruminococcus, Streptococcus*	Glutamate II synthesis
Glutamate synthase gltD	11	*Anaerostipes, Bacillus, Bifidobacterium, Blautia, Clostridium, Escherichia, Lactobacillus, Listeria, Roseburia, Ruminococcus, Streptococcus*	Glutamate II synthesis
Glutamine synthetase	17	*Bacteroides, Bifidobacterium, Citrobacter, Clostridium, Coprococcus, Enterobacter, Enterococcus, Escherichia, Eubacterium, Faecalibacterium, Klebsiella, Lactobacillus, Proteus, Providencia, Roseburia, Ruminococcus, Streptococcus*	L-glutamine formation
Glutathione reductase	13	*Bifidobacterium, Citrobacter, Clostridium, Enterobacter, Enterococcus, Escherichia, Faecalibacterium, Klebsiella, Lactobacillus, Listeria, Proteus, Providencia, Streptococcus*	Glutathione degradation
Glutathione S-transferase	10	*Citrobacter, Enterobacter, Enterococcus, Escherichia, Klebsiella, Lactobacillus, Oxalobacter, Proteus, Providencia, Streptococcus*	Glutathione degradation
Glutathione synthetase	12	*Citrobacter, Clostridium, Enterobacter, Enterococcus, Escherichia, Klebsiella, Lactobacillus, Listeria, Pediococcus, Proteus, Providencia, Streptococcus*	Glutathione synthesis
Glycine amidinotransferase	1	*Streptomyces*	GABA degradation
Histidine ammonia-lyase	20	*Acidaminococcus, Actinomyces, Alistipes, Bacteroides, Bifidobacterium, Blautia, Citrobacter, Clostridium, Enterobacter, Enterococcus, Escherichia, Eubacterium, Klebsiella, Lactobacillus, Odoribacter, Prevotella, Providencia, Providencia, Roseburia, Streptococcus*	Histamine degradation
Histidine decarboxylase	13	*Bifidobacterium, Citrobacter, Clostridium, Eggerthella, Enterobacter, Gordonibacter,*	Histamine synthesis

		Klebsiella, Lactobacillus, Morganella, Staphylococcus, Streptococcus	
Kynurenine formamidase KynB	1	*Klebsiella*	Degradation of tryptophan to kynurenine
Lactocepin	2	*Lactobacillus, Lactococcus*	Degradation of proinflammatory chemokine IP-10
Lactoyl-CoA dehydratase	3	*Clostridium, Coprococcus, Megasphaera*	Propionic acid synthesis
Methylmalonyl-CoA decarboxylase	29	*Actinomyces, Alistipes, Bacteroides, Bifidobacterium, Blautia, Butyrivibrio, Clostridium, Dialister, Dorea, Eubacterium, Holdemania, Lactobacillus, Megasphaera, Odoribacter, Parabacteroides, Phascolarctobacterium, Prevotella, Roseburia, Streptococcus, Veillonella*	Propionic acid synthesis
Propionaldehyde dehydrogenase	15	*Blautia, Citrobacter, Clostridium, Dorea, Enterobacter, Enterococcus, Eubacterium, Lactobacillus, Listeria, Roseburia, Ruminococcus, Streptococcus*	Propionic acid synthesis
L-aspartate oxidase	10	*Citrobacter, Clostridium, Enterobacter, Escherichia, Faecalibacterium, Klebsiella, Oxalobacter, Proteus, Providencia, Streptococcus*	N-acetyl aspartate degradation
Linoleic acid isomerase	23	*Actinomyces, Alistipes, Anaerostipes, Bacteroides, Bifidobacterium, Blautia, Butyrivibrio, Carnobacterium, Clostridium, Eggerthella, Enterococcus, Eubacterium, Gordonibacter, Helicobacter, Holdemania, Lactobacillus, Lactococcus, Listeria, Roseburia, Ruminococcus*	Linoleic acid conjugation
Microbial anti-inflammatory molecule	1	*Faecalibacterium*	Inhibition of transcription factor (NF)-kB and immune response of t-lymphocytes Th-1 and Th-2
Monoamine oxidase	5	*Enterobacter, Escherichia, Klebsiella, Proteus, Pseudomonas*	Serotonin, dopamine and norepinephrine degradation
Myo-inositol 2-dehydrogenase	13	*Anaerostipes, Bacteroides, Bifidobacterium, Blautia, Clostridium, Enterobacter, Enterococcus, Klebsiella, Lactobacillus, Listeria, Ruminococcus, Streptococcus*	Inositol degradation
Myo-inositol-1(or 4)-monophosphatase	10	*Acinetobacter, Bacteroides, Bifidobacterium, Enterobacter, Enterococcus, Escherichia, Lactobacillus, Listeria, Streptococcus*	Inositol synthesis

Myo-inositol-1-phosphate synthase	1	*Streptomyces*	Inositol
Nitric oxide dioxygenase	10	*Bacillus, Citrobacter, Clostridiales, Enterobacter, Escherichia, Klebsiella, Listeria, Proteus, Providencia, Streptococcus*	Nitric oxide degradation
Nitric oxide reductase NorB	2	*Enterobacter, Pseudomonas*	Nitric oxide degradation
Nitric oxide reductase NorC	2	*Enterobacter, Pseudomonas*	Nitric oxide degradation
Nitric oxide synthase	6	*Bacillus, Geobacillus, Listeria, Staphylococcus, Streptococcus, Streptomyces*	Nitric oxide synthesis
Ornithine carbamoyltransferase	5	*Escherichia, Megamonas, Mitsuokella, Moritella, Veillonella*	Arginine synthesis pathway in prokaryotes
Phenylalanine aminotransferase	3	*Actinomyces, Bifidobacterium, Streptococcus*	Phenylalanine synthesis
Phenylalanine-specific permease	6	*Citrobacter, Enterobacter, Escherichia, Klebsiella, Proteus, Providencia*	Phenylalanine transportation
Phenyllactate dehydratase	9	*Bacteroides, Blautia, Butyrivibrio, Clostridium, Coprococcus, Dorea, Eubacterium, Lactobacillus, Roseburia*	Indole-3-propionic acid formation
Phenyllactate dehydrogenase	7	*Bifidobacterium, Clostridium, Enterococcus, Eubacterium, Roseburia, Ruminococcus*	Formation of 4-hydroxyphenyl pyruvate from prephenate, part of phenylalanine and tyrosine synthesis metabolic pathway
Phosphotransacetylase	43	*Actinomyces, Alistipes, Anaerostipes, Bacillus, Bacteroides, Bifidobacterium, Blautia, Butyrivibrio, Citrobacter, Clostridium, Corynebacterium, Desulfovibrio, Dorea, Eggerthella, Enterobacter, Enterococcus, Escherichia, Eubacterium, Gordonibacter, Helicobacter, Lactobacillus, Listeria, Megasphaera, Odoribacter, Parabacteroides, Prevotella, Proteus, Providencia, Ruminococcus, Streptococcus*	Acetic acid synthesis
Phosphotransbutyrylase	3	*Blautia, Clostridium, Streptococcus*	Formation of outer cell d-(−)-3-hydroxybutyric acid
Prephenate dehydrogenase	10	*Citrobacter, Enterobacter, Enterococcus, Escherichia, Lactobacillus, Lactococcus, Listeria, Proteus, Providencia, Streptococcus*	Tyrosine synthesis form prephenate
Pyruvate dehydrogenase aceE	8	*Citrobacter, Enterobacter, Escherichia, Helicobacter, Klebsiella, Oxalobacter, Proteus, Providencia*	Pyruvic acid synthesis out of glucose
Pyruvate dehydrogenase aceF	6	*Citrobacter, Enterobacter, Escherichia, Klebsiella, Proteus, Providencia*	Pyruvic acid synthesis out of glucose

Pyruvate kinase pykA	6	*Citrobacter, Enterobacter, Escherichia, Klebsiella, Proteus, Providencia*	Pyruvic acid synthesis out of glucose
Pyruvate kinase pykF	13	*Bacteroides, Bifidobacterium, Citrobacter, Clostridium, Enterobacter, Escherichia, Eubacterium, Klebsiella, Lactobacillus, Proteus, Providencia, Roseburia, Ruminococcus*	Pyruvic acid synthesis out of glucose
Quinolinate synthase	10	*Citrobacter, Clostridium, Enterobacter, Escherichia, Faecalibacterium, Klebsiella, Oxalobacter, Proteus, Providencia, Streptococcus*	Participation in degradation of quinolinic acid to nicotinamide adenine dinucleotide (NAD)
Serine hydroxymethyltransferase	24	*Alistipes, Anaerostipes, Bacteroides, Bifidobacterium, Blautia, Citrobacter, Clostridium, Desulfovibrio, Dorea, Enterobacter, Enterococcus, Escherichia, Eubacterium, Faecalibacterium, Helicobacter, Klebsiella, Lactobacillus, Listeria, Oxalobacter, Proteus, Providencia, Roseburia, Ruminococcus, Streptococcus*	Glycine formation from serine
Serine racemase	3	*Enterococcus, Listeria, Streptococcus*	Formation of D-serine from L-serine
Serotonin N-acetyltransferase	24	*Anaerostipes, Blautia, Butyrivibrio, Butyrivibrio, Clostridium, Desulfovibrio, Dialister, Dorea, Enterococcus, Eubacterium, Lactobacillus, Megasphaera, Roseburia, Ruminococcus, Streptococcus*	Serotonin degradation for melatonin formation
Serpin	2	*Bifidobacterium*	Inhibitor of pancreatic and neutrophilic elastase during inflammation
Spermidine synthase	26	*Bacteroides, Blautia, Butyrivibrio, Citrobacter, Clostridium, Enterobacter, Enterococcus, Escherichia, Eubacterium, Megasphaera, Peptoclostridium, Roseburia, Ruminococcus, Streptococcus*	Spermidine synthesis
Tryptophan 2,3-dioxygenase	3	*Enterobacterales, Klebsiella, Streptococcus*	Degradation of tryptophan to kynurenine
Tryptophan permease	3	*Citrobacter, Enterobacter, Escherichia*	Tryptophan transportation
Tryptophan-specific transport protein	4	*Citrobacter, Enterobacter, Escherichia, Klebsiella*	Tryptophan transportation
Tryptophan synthase alpha	13	*Anaerostipes, Bacteroides, Bifidobacterium, Blautia, Citrobacter, Clostridium, Enterobacter, Enterococcus, Escherichia, Eubacterium, Lactobacillus, Proteus, Roseburia*	Tryptophan synthesis

Tryptophan synthase beta	13	*Anaerostipes, Bacteroides, Bifidobacterium, Blautia, Citrobacter, Clostridium, Enterobacter, Enterococcus, Escherichia, Eubacterium, Lactobacillus, Proteus, Roseburia*	Tryptophan synthesis
Tryptophanase	7	*Alistipes, Bacteroides, Citrobacter, Clostridium, Enterobacter, Escherichia, Proteus*	Synthesis of indole from tryptophan
Tyrosine aminotransferase	6	*Citrobacter, Enterobacter, Escherichia, Klebsiella, Proteus, Providencia*	Tyrosine synthesis
Tyrosine decarboxylase	12	*Bifidobacterium, Clostridium, Dialister, Enterococcus, Escherichia, Eubacterium, Lactobacillus, Peptoclostridium, Streptococcus*	Synthesis of tyramine and dopamine
Tyrosine-specific transport protein	6	*Citrobacter, Enterobacter, Escherichia, Klebsiella, Proteus, Providencia*	Tyrosine transportation
Vinylphenol reductase	7	*Bacteroides, Blautia, Butyrivibrio, Clostridium, Collinsella, Eubacterium, Lactobacillus*	4-Ethyl phenol formation
